# Involvement of the FAK Network in Pathologies Related to Altered Mechanotransduction

**DOI:** 10.3390/ijms21249426

**Published:** 2020-12-10

**Authors:** Enrica Urciuoli, Barbara Peruzzi

**Affiliations:** Multifactorial Disease and Complex Phenotype Research Area, Bambino Gesù Children’s Hospital, IRCCS, 00165 Rome, Italy; enrica.urciuoli@opbg.net

**Keywords:** Focal Adhesion Kinase (FAK), focal adhesions, mechanotransduction, tissue matrix stiffness, extracellular matrix

## Abstract

Mechanotransduction is a physiological process in which external mechanical stimulations are perceived, interpreted, and translated by cells into biochemical signals. Mechanical stimulations exerted by extracellular matrix stiffness and cell–cell contacts are continuously applied to living cells, thus representing a key pivotal trigger for cell homeostasis, survival, and function, as well as an essential factor for proper organ development and metabolism. Indeed, a deregulation of the mechanotransduction process consequent to gene mutations or altered functions of proteins involved in perceiving cellular and extracellular mechanics can lead to a broad range of diseases, from muscular dystrophies and cardiomyopathies to cancer development and metastatization. Here, we recapitulate the involvement of focal adhesion kinase (FAK) in the cellular conditions deriving from altered mechanotransduction processes.

## 1. Introduction

Eukaryotic cells are able to sense extracellular mechanical stimuli and translate them into chemical signals by a phenomenon known as mechanotransduction. The relevance of mechanical stimulation on cell functions has been historically investigated in tissues mainly subjected to macroscopic external mechanical loads, such as bones and muscles. Indeed, every cell of our body is surrounded by a matrix with a peculiar stiffness, which is strictly related to the cell physiological/pathological condition. Cell adhesion on tissue matrix exerts a key pivotal role in proper metabolism, protein synthesis, and cell survival, and those proteins able to perceive and transduce the mechanical signals are identified as mechanosensors. Mechanosensors are a heterogeneous category of proteins ranging from the plasma membrane channels sensitive to mechanical stretch to cytoplasmic and cytoskeletal proteins, as well as nuclear structural proteins, being the nucleus itself subjected to conformational changes in response to force.

Recent findings concerning a deregulation in the mechanotransduction machinery have shed light on the strict link between cell mechanics and cell homeostasis. In particular, some diseases [1,2,3] are known to give rise to, or arise from, changes in the cell mechanical and structural properties, which disrupt the cell physiological functionality.

## 2. FAK and Mechanotransduction

**Figure 1:**
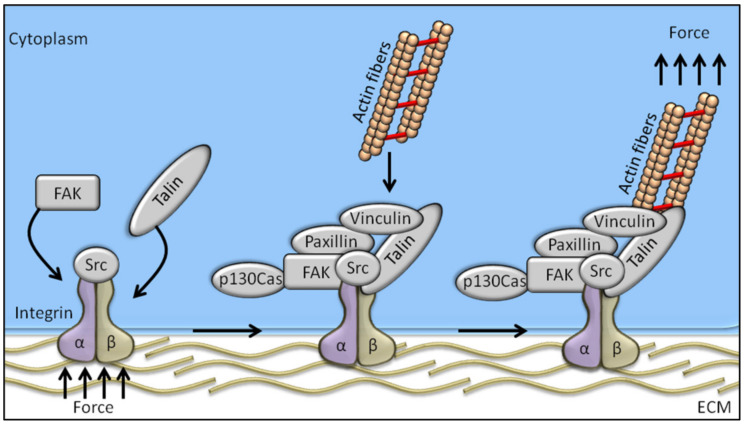
Schematic representation of the mechanosensing machinery at the focal adhesion site. Changes in ECM stiffness, shear stress or other mechanical stimuli are sensed by cells through the integrin pathway whose activation recruits FAK. The integrated activation of talin, vinculin, paxillin, and p130Cas drives the transfer of the mechanical signal from integrins to the actin cytoskeleton.

Among the proteins known to be involved in the mechanotransduction process, integrins are likely the best representatives of mechanosensors. They connect the cytoskeleton to the extracellular matrix (ECM), thereby linking the cellular transcriptional machinery to its external mechano-environment [4]. Integrins are known to regulate a large number of intracellular signalling pathways involving cytoplasmic kinases, small GTPases, and scaffolding proteins, and modulate the activity of other receptors at cell surface level [4,5]. FAK (focal adhesion kinase), a non-receptor tyrosine kinase predominantly localized at the focal adhesions of adherent cells [6,7], is likely the earliest and most important component of integrin signalling to be identified. Studies conducted in the early 1990s on the viral oncogene v-Src identified FAK as one of the substrates of tyrosine-kinase activity [8], and FAK tyrosine phosphorylation is the first event occurring in response to integrin-mediated cell adhesion [9]. Cells adhering on a stiff matrix usually develop discrete multiprotein complexes at the inner site of the cell membrane, known as focal adhesions (FAs). FAs are the main component in the interaction between the extracellular matrix and cells, and are responsible for sensing and translating mechanical stimuli arising from the ECM to the cellular cytoskeleton. Focal adhesions are assembled as complex structures comprising both transmembrane and intracellular layers. Proteins composing the intracellular layer of FAs are known to connect integrins (transmembrane molecules directly in contact to the ECM) with the actin cytoskeleton. The molecular composition of FA structures can greatly vary and is extremely sensitive to ECM mechanics and composition. Integrin heterodimers, composed by α- and β-subunits, govern the affinity of the assembled receptor for different ECM components, its cell type specificity, and the biophysical mechanisms of FAK activation [10]. Regarding the proteins comprised in the intracellular FA layer, some of them are mechano-responsive factors, while others have been recognized as outside-in signal transduction [11] (Figure 1).

The adapter protein talin is involved in linking the intracellular domain of β-integrin to the F-actin, thereby serving as a bridge between membrane signals and the cellular cytoskeleton [12]. Talin’s globular head domain and flexible rod domain contain several binding sites for vinculin, integrin, and actin [13], and talin activation requires two monomers form an antiparallel dimer [14]. The content of vinculin at the focal adhesions directly correlates with the force applied to the focal adhesion, and is also involved in transmitting force inside-out by increasing the binding of integrin–talin complexes to the ECM via its head domain, while the tail domain is needed to propagate the mechanical load to the actin cytoskeleton [15]. Paxillin is a docking protein mainly localized in the intracellular layer of focal adhesion sites. The main role exerted by paxillin is to link together structural and signalling partners [16], which is achieved by different interacting domains, conferring paxillin high-affinity binding properties [16]. Indeed, paxillin is involved in binding activated vinculin and paxillin LD motif-binding protein (actopaxin), once phosphorylated by FAK or Src, thus stabilizing the FA–cytoskeleton interaction [17]. P130Cas adaptor protein, a stretch-sensitive factor, has been recently proposed as a mechanosensor [18]. Integrin activation induces p130^Cas^ recruitment to the focal adhesions, where it switches to an unfold conformation by exposing tyrosine residues to be phosphorylated. Once phosphorylated, p130Cas triggers several signalling pathways following mechanical stimulation, thereby representing a key pivotal factor in the mechanotransduction machinery.

Among the aforementioned proteins involved in mechanotransduction, FAK is one of the first molecules recruited to focal adhesions in response to external mechanical stimuli. The FAK-dependent cascade signalling starts with FAK activation by autophosphorylation on the Tyr-397 residue, which represents the cell mechanotransduction trigger. More in detail, Bauer et al. showed that FAK could be directly activated by mechanical forces applied at the focal adhesion, by inducing conformational changes in the FAK structure and the consequent trigger in focal adhesion-mediated signals [19]. The need of living cells to maintain and control tension at critical sites and to transduce force to the nucleus is guaranteed by the relationship between FAK and the contractile cytoskeleton [20]. For example, local remodelling of cytoskeleton and nucleus compression, required for cell polarization, depends on site-specific FAK activation [21]. Taken all together, these data clearly recognize FAK tyrosin kinase as a homeostatic mechanosensor that spontaneously self-adjusts to reach a state where its activation matches the ECM stiffness [22].

## 3. FAK Alterations in Pathologies Associated with Altered Mechanotransduction

Due to the pivotal role played by FAK in the integration between external mechanical stimulation and the intracellular mechanotransduction machinery, it is not surprising that alterations of FAK functionality are strictly related to deregulated cell mechanics. In particular, here we describe how FAK activation and function is regulated by ECM stiffness and promotes the fibrotic and inflammatory events associated with pathological conditions, and describe its involvement in the dysfunction of mechanotransduction of endothelial, cardiac muscle, and bone cells.

### 3.1. FAK Network and ECM Stiffness

Focal adhesion assembly/disassembly and integrin-mediated mechanotransduction respond to ECM stiffness. In mammary tissue, kidney, and liver, integrins and FA complexes are rarely expressed if not absent, whereas high levels of these proteins are detected in cell lines and primary cells cultured in vitro on plastic surfaces [23], suggesting that integrin/FA activation and clustering is induced by increasing matrix stiffness [24,25]. In particular, the study by Yeh et al. [26] revealed that β1-integrin expression is strictly related to ECM stiffness. Indeed, β1 integrin protein content is selectively downregulated on a soft matrix by endocytosis and lysosomal degradation, as well as Caveolin-1 (Cav-1) is down-regulated in relationship to declining matrix stiffness. Cav-1, the caveolae/lipid rafts structural protein, is strictly related to mechanotransduction, being involved in integrin-dependent signalling [27] and FA assembly/turnover [28], and acting as a mechanosensor in mechanotransduction [29]. With Cav-1 activation being mediated by adhesion signals through FAK or Src activity [30,31], Yeh et al. demonstrated that treating mouse mammary gland epithelial (NmuMG) cells with a β1-integrin blocking antibody, as well as with the FAK inhibitor PF-573228, reduced the collagen-induced activation of Cav-1. These findings identified β1 integrin-mediated adhesion as the upstream signal driving Cav-1 activatory phosphorylation through FAK activity. Indeed, the study by Yeh et al. demonstrates that integrin binding to the ECM causes a receptor conformational change and activation of FAK/Src-involving pathways in cells grown on a stiff matrix. The following FAK and Src activation increases Cav-1 phosphorylation and membrane stability of lipid rafts, which, in turn, provides inside-out signals to support the clustering of β1 integrin, thereby promoting focal adhesion assembly and cytoskeleton organization.

In the context of cell mechanostranduction, recent evidence highlighted the crucial role of the nucleus and its structural proteins in sensing and responding to the ECM stiffness. Indeed, the expression of lamin A/C, an intermediate filament of the nucleoskeleton, has been found to correlate with tissue stiffness, being highly expressed in cells seeded onto a stiff matrix and down-regulated in cells grown on a soft matrix [32]. Accordingly, lamin A/C takes part in several physiological and pathological processes, such as cell differentiation and function [32] and cancer development [33,34]. Among its several activities, FAK has been found to be present in the nucleus and regulate gene expression by influencing tumorigenesis [35,36]. As for its nuclear activity, we hypothesised a further interaction between FAK and Src tyrosine kinase in addition to that at the focal adhesions, especially in bone cancer cells [37,38]. Moreover, FAK has been demonstrated to be involved in the regulation of nuclear deformity and cellular senescence; indeed, Chuang et al. [39] demonstrated that FAK pharmacological inhibition by PF-573228 treatment induced a senescence-like pattern in lung cancer cells, leading to a reduction in lamin A/C expression and to p53 up-regulation. This result suggests that the expression of p53 and the maintenance of lamin A/C levels are associated with FAK signalling to shape regular nuclear morphology and act as a anti-senescence signal. Moreover, these authors also demonstrated that the activation of the cell senescence program is achieved by FAK signalling inhibition, thereby providing a new therapeutic approach to limit tumour growth [39].

### 3.2. FAK in Fibrosis and Inflammation

The pathogenesis of fibrosis, an exuberant fibroproliferation after injury, has traditionally been explained by the cytokine-based paradigm, in which the involvement of ECM- and mechanical-load-mediated effects have been largely overlooked. Indeed, studies by Gurtner’s group shed light on the link between mechanical cues and fibrosis/inflammation in a process largely mediated by FAK activity [40,41]. Through a genome-wide microarray analysis on wild-type mouse scars subjected to skin tension, Wong et al. [41] found that FAK is activated by cutaneous injury and that mechanical force substantially potentiated this effect, thereby proving that both inflammation and fibrosis are mediated by FAK activity. Fibroblast-specific FAK knock-out mice have substantially less inflammation and fibrosis than control mice in a model of hypertrophic scar formation. In this model, FAK acts through ERK (Extracellular-Related Kinase) to mechanically trigger the secretion of the monocyte chemoattractant protein-1 (MCP-1), a potent chemokine that is linked to human fibrotic disorders. FAK inhibition by small molecule PF573228 attenuated MCP-1 signalling and inflammatory cell recruitment, thereby blocking the aforementioned effects in human cells, and reducing scar formation in vivo. All together, these data show that mechanical stimuli regulate fibrosis through inflammatory FAK–ERK–MCP-1 pathways, and that molecular strategies targeting FAK can effectively uncouple physical force from pathologic scar formation [41]. FAK is also a key factor in fibroblast migration, as demonstrated by Wang et al.: FAK-null fibroblasts showed a reduction in migration speed and directionality, and an impaired response to mechanical stimulation in comparison to normal cells with wild-type FAK [42]. These data suggest that FAK is strongly involved in the response of migrating cells to mechanical stimulation, and that this response is mediated, at least in part, by FAK activating phosphorylation at Tyr-397.

Gurtner’s group also demonstrated the crucial role of FAK in keratinocyte fibrogenic gene expression, providing further evidence that altered mechanotransduction pathways are linked to both inflammation and fibrosis, and that FAK is a key mediator of these processes [43]. More in detail, keratinocytes collected from wild-type and conditional keratinocyte-specific FAK-deleted mice were sorted into single cells and assessed by a microfluidic-based platform for high-resolution transcriptional analysis. The authors demonstrated that FAK-deleted keratinocytes showed differential expression of the genes involved in mechanotransduction, including ECM genes, genes regulating cell–ECM adhesion and ECM-mediated mechanotransduction, and factors involved in tissue repair and matrix remodelling, such as Collagen type IV (Col4) subunits and Keratin 6 (Krt6), FAK, CD44, Paxillin, integrins αv and β1, β4, β6, and β8, and matrix metalloproteinases (MMPs) and tissue inhibitors of MMPs (TIMPs) [43], respectively.

These findings indicate that mechanical force regulates fibrosis through inflammatory pathways involving FAK activity, and that targeting inhibition of FAK can effectively uncouple mechanical force from pathologic scar formation.

### 3.3. FAK Involvement in Endothelial Cell Mechanotransduction and Function

In systemic circulation, blood is pumped through the vessels in a pulsatile fashion determined by the heart contractile activity; for this reason, blood vessels are sensitive to stretch and shear stress mechanical stimulation [44]. Smooth muscle cells and endothelial cells of the arterial wall sense and respond to these stimulations by adapting their cytoskeleton to accommodate to the force exerted on the vessel. Vascular cells are subjected to several mechanical forces generated by gravity, tension, compression, hydrostatic pressure, and fluid shear stress [45], which modulate FAK activity, interactions through phosphorylation, and its subcellular localization in the cell. Once activated by a mechanical load, FAK, affecting focal adhesion dynamics and localization, controls directional endothelial cell migration and maintains cell polarity, modulating cell proliferation depending on the physiological cell state and suppressing caspase-mediated apoptosis, regulating angiogenesis, endothelial activation, leukocyte adhesion, vascular inflammation, and the vascular endothelial barrier [46].

The relevance of matrix stiffness in cancer-related pathological conditions is highlighted by the evidence that solid tumours are typically stiffer than healthy normal tissue [47]. Moreover, an increase of matrix stiffness in the tumour microenvironment is correlated with poor prognosis [48,49], with an increase in tumour metastatization and with a characteristic tumour phenotype of vascularisation (disruption of vessel integrity and increase in endothelial permeability) [50]. FAK, together with Src, is involved in the regulation of vascular permeability [51], and, as previously reported, its phosphorylation is related to tumour stiffening and progression and to poor patient prognosis [52]. In the study by Wang et al., FAK activation was found to exert a pivotal role in the regulation of vascular integrity mediated by matrix stiffness [53], since FAK inhibition by PF-573228 treatment avoids the increase in vascular permeability induced by matrix stiffness both in vitro and ex ovo chicken embryo culture systems. Moreover, FAK inhibition prevents Src translocation to the cell membrane and VE-cadherin activation induced by enhanced matrix stiffness, further demonstrating the key role of FAK in the regulation of endothelial integrity dependent on matrix stiffness.

### 3.4. FAK in Heart Disease

A fully developed adult heart responds to sustained hemodynamic overload by cardiac hypertrophy, an adapting process that if prolonged, leads to arrhythmia, congestive heart failure, and sudden death. Changes in cytoskeletal structures of cardiac muscle cells can induce abnormal hypertrophy and are associated with patients’ poor prognosis. Although several systemic factors such as vasoactive peptides, catecholamines, cytokines, and growth factors are involved in the development of cardiac hypertrophy, stimuli of mechanical nature have been identified as the main signal to trigger hypertrophy in the overloaded myocardium [54,55]. Several studies have suggested that cardiomyocyte hypertrophy is regulated by biochemical pathways dependent on FAK, which constitutes, once again, an important factor downstream to mechanical stimuli. Indeed, both cardiomyocytes subjected to pulsatile mechanical stretch and overloaded feline and rat myocardium are known to induce FAK activation [56,57]. Besides its critical roles in vitro and consistently with them, the constitutive deletion of the FAK gene in mice resulted in embryonic lethality, mainly due to defects in the axial mesoderm and cardiovascular system [58], with absence of a normal heart and fully developed blood vessels. Starting from these data, the crucial role of FAK in heart development and function has been hypothesised, but the embryonic lethality of FAK total knock-out mice precluded further studies in adults’ hearts. To overcome this, Peng et al. devised a mouse model in which FAK is selectively inactivated in cardiomyocytes (CFKO) [59]. In these animals, authors described the development of an eccentric cardiac hypertrophy due to pressure overload achieved by transverse aortic constriction. Moreover, they also showed an increase in heart/body weight ratio, an increase in cardiac hypertrophy markers, the presence of multifocal interstitial fibrosis, and an enhancement of collagen I and VI expression in CFKO mice compared with control animals, thereby confirming the involvement of FAK in the regulation of adult heart hypertrophy in vivo.

In support of this, many other works have confirmed the strict relationship between FAK, mechanical stimulation, and heart development and function. The spatial distribution of FAK in cardiomyocytes varies significantly in response to mechanical stress, preferentially overlapping with the I-band rather the A-band in mechanically stimulated cardiomyocytes. This outcome suggests that signals of mechanical nature govern the relocation of FAK to distinct sites of cardiomyocytes [60]. It is worth noting that prolonged stimulation of cardiomyocytes by mechanical stress induces a FAK nuclear localization, whose function is still unclear, but it has been hypothesized to regulate chromatin structure, transcription, mRNA processing, and nuclear export [61].

Taken together, these findings demonstrate that myocardium cells have an intrinsic and physiologic ability to perceive and respond to mechanical stimulation through the activity of the FAK mechanosensor, and that prolonged and abnormal loading conditions can lead to maladaptive heart remodelling processes, causing deregulated physiological functions, such as development of pathological cardiac hypertrophy and heart failure.

### 3.5. FAK-Mediated Mechanotransduction in Bone Cells

Skeleton stimulation by mechanical loading is crucial for maintaining bone mass, strength and functions. A decrease in mechanical load due to muscle paresis, prolonged immobilization, or weightlessness during space flights causes bone loss, while enhanced loading stimulations favour bone formation [62]. Moreover, the mechanical forces acting on the bone marrow are crucial for the differentiation fate of mesenchymal stromal cells (MSCs). Indeed, the work by Discher’s group clearly demonstrated that MSCs grown on a stiff matrix are physiologically driven towards osteogenic differentiation, while MSCs on softer matrices spontaneously differentiated into adipocytes [32]. These data clearly demonstrate the strict relationship between the cell’s behaviour and functionality and the ECM to which it is attached. Leucht et al. exploited the peculiar features of the bone marrow cavity microenvironment to shed light on the mechanisms driven by mechanical stimulations that orchestrate the integrated and ultimately synchronized tissue-level response in a living organism [63]. Indeed, stem cells within the bone marrow are maintained in a quiescent state and become mobilized in response to several signalling pathways, some of which depend on physical stimuli. By exploiting a small pinhole in the tibia cortex and the application of a permanent device able to create a short distance into the bone marrow cavity producing a mechanical stimulation, Leucht et al. demonstrated that the site-specific physical stimulus triggered the expression of Sox9 and Runx2, two master genes that function as transcription factors and are required for the differentiation of stem cells towards an osteogenic lineage. This mechanical force produced a specific strain that is fine-tuning associated with the arrangement and orientation of newly deposited type I collagen fibrils in the bone marrow cavity. To further examine this phenomenon, authors conditionally inactivated FAK in the osteoblast lineage and demonstrated that these cells were completely disabled to respond to physical stimuli. Indeed, up-regulation of osteogenic genes upon mechanical stimulation, disorganized collagen fibrils deposition, and total lack of the mechanically-induced osteogenic response were no longer reported. Collectively, these data provide in vivo evidence for the basis of mechanotransduction in the bone marrow cells and the effects of physical stimuli detected by skeletal progenitor cells.

In the same context of bone microenvironment, Pilz’s group showed a correlation between FAK and protein-kinase G in the regulation of physiological osteoblast mechanotransduction [64]. Mechanical stimulation of a long bone segment induced interstitial fluid flow acting on osteoblasts such as fluid shear stress that rapidly enhances the intracellular calcium concentration and nitric oxide (NO) synthesis, and activates protein kinase Akt-dependent osteoblast proliferation and survival. The increase in the intracellular calcium concentration induced independent activation of both FAK and PKG protein kinases, which cooperatively converged on Src tyrosine kinase, responsible in activating the Akt/GSK3/β-catenin signalling in shear-stressed osteoblasts. These data proved that osteoblasts’ mechanical stimulation acts on growth regulatory pathways essential for maintaining skeletal integrity.

Other than osteoblasts, the maintenance of bone tissue homeostasis depends on the regulated differentiation and activity of bone-resorbing osteoclasts [65,66], while osteoclast dysfunction, mainly hyperactivation, is associated with several pathological conditions of bone tissue, such as osteoporosis and cancer-related bone metastases [67]. Indeed, Bouton’s group demonstrated that FAK family kinases (FAK and Pyk2 tyrosine kinases) are involved in the regulation of osteoclast structure and function. The authors generated a conditional knock-out mouse model in which FAK expression was selectively deleted in cells of the myeloid lineage, where osteoclasts developed [68]. Through this model, Bouton’s group demonstrated that FAK^Δmyeloid^ mice had no significant defects in bone volume or architecture in comparison with control littermates, likely explained by the compensatory effect mediated by Pyk2. In in vitro experiments performed by collecting osteoclast precursors from FAK^Δmyeloid^ mice, osteoclast-mediated bone resorption was reduced by 30%, and an impaired signalling through the Macrophage Colony-Stimulating Factor (M-CSF) receptor (CSF-1R) was described. To demonstrate that Pyk2 might functionally compensate FAK deletion, Bouton et al. showed that the combined depletion of FAK and Pyk2 inhibited the differentiation of bone marrow precursors into multinucleated osteoclasts. Moreover, in FAK/Pyk2-depleted mice, a marked deregulated morphology was observed, mainly in the podosomes, which are the structures formed by osteoclasts at the site of contact with ECM and are crucial for proper bone resorption [69]. These data clearly support the notion that small molecule inhibitors targeting tyrosine kinases, such Src and FAK/Pyk2, are good candidate treatments for bone metastatic disease [70], and that proper mechano-environment sensing and mechanotransduction by osteoclasts is crucial for their function and for bone tissue homeostasis.

## 4. Therapeutic Implications for Targeting Mechanoactivator FAK

As discussed throughout this review, tyrosine kinase FAK plays pivotal roles in the physiological response to extracellular mechanical stimuli, and its activity becomes impaired in pathological conditions associated with altered mechanotransduction events. For these reasons, therapeutic approaches aimed at targeting FAK activity may be good candidates to counteract the deleterious effects of altered mechanotransduction-related pathologies. Indeed, several pathological conditions are associated with a stiffening of the surrounding ECM, leading to a hyper-activation of the cellular mechanosensing machinery, in which FAK becomes dramatically activated.

A clear example of this phenomenon is represented by cancer progression, especially in breast cancer, whose first detection is often achieved through breast palpation aimed at identifying a fibrotic focus with increased stiffness compared with surrounding tissue.

In this context, the inhibition of FAK has been identified as a promising and effective therapeutic approach based on blocking the activation of tumour-favouring signals downstream extracellular mechanical stresses. Besides the aforementioned FAK inhibitors used in in vitro experiments on cell cultures, several compounds are currently under clinical and preclinical investigation in order to identify promising drugs for human diseases [71,72]. To completely inhibit FAK activity, the ideal drug should have a dual specificity, for FAK and for protein tyrosine kinase 2β (Pyk2), a FAK homologous that can compensate its activity. Indeed, the specificity only towards FAK of the two early inhibitors dramatically reduced the effectiveness of these drugs in reducing proliferation and inducing apoptosis in cancer cells, likely due to the redundant and not fully inhibited activity of Pyk2 [73,74]. One of the first clinically available dual-specific inhibitor was PF-562271, an ATP-competitive reversible inhibitor of the catalytic activity for FAK and Pyk2 that was effective in inducing tumour regression [75] and in reducing tumour growth, invasion, and metastatisation [76] in preclinical studies on mouse xenograft models. Moreover, PF-562271 was evaluated in a phase 1 clinical trial on patients with advanced solid cancers, demonstrating no significant side effects, and maintaining a stable and not progressive disease in about one third of patients [77]. Among the FAK inhibitors developed in the last decades, a very promising drug is NVP-TAE226, showing a potent anti-tumoral effect both in in vitro and in vivo preclinical studies on several solid cancers [78,79,80]. However, its evaluation in clinical trials was hindered by its impairment of glucose metabolism as a side effect. A recently developed FAK inhibitor, VERSUS-4718, has been reported to reduce tumour-associated fibrosis and tumour progression in an in vivo model of human pancreatic adenocarcinoma [81].

All these data provide strong evidence that targeting activators of the mechanotransduction process could be instrumental in reverting or, at least, limiting fibrosis and/or ECM stiffening associated with several pathologies.

## 5. Conclusions

Every cell in our tissues and organs is physiologically exposed to stimulation of mechanical nature, and can translate these signals into biochemical information through a fine-tuning regulated process known as mechanotransduction. This process is involved in the regulation of several cellular functions, such as cell adhesion, migration, proliferation, and survival, as well as in the progression of diseases such as cancer. Focal adhesions are the main sites in the cell in which mechanotransduction is triggered following the interactions between extracellular mechanical environments and intracellular biochemical signalling molecules. As the first recruited and key component of focal adhesions, FAK has been suggested to play important roles in the mechanotransduction process and, if deregulated, in the resulting pathologies. Here, we reviewed the current molecular evidence about FAK-dependent mechanotransduction, with a special focus on those pathological conditions mediated by mechano-environment alteration and involving FAK activity, thereby providing a further rationale about the effectiveness of FAK pharmacological inhibition.

## Abbreviations

CAV	Caveolin-1
CSF-1R	Colony Stimulating Factor 1 Receptor
ECM	Extracellular Matrix
ERK	Extracellular-Related Kinase
FA	Focal Adhesion
FAK	Focal Adhesion Kinase
FSS	Fluid Shear Stress
MCP-1	Monocyte Chemoattractant Protein-1
M-CSF	Macrophage Colony-Stimulating Factor
MSCs	Mesenchymal Stromal Cells
NO	Nitric Oxide
PKG	Protein-Kinase G
Pyk2	Protein Tyrosine Kinase 2β
Runx-2	Runt-Related Transcription Factor 2
Sox9	SRY-Box Transcription Factor 9
VE-cadherin	Vascular Endothelial Cadherin
